# Supplementary material for: Potential Markers for Selecting Self-Eliminating Apple Genotypes
Source: Plants (Basel). 2021 Aug 5;10(8):1612. doi: 10.3390/plants10081612 (PMC8398410; doi:10.3390/plants10081612)
Supplement: Supplementary file 1 [file plants-10-01612-s001.zip › plants-1275088-supplementary.pdf]

plants

Article

Potential Markers for Selecting Self-Eliminating Apple Genotypes

Aurelijus Starkus, Birute Frercks *, Dalia Gelvonauskiene, Ingrida Mazeikiene, Rytis Rugienius, Vidmantas Bendokas and Vidmantas Stanys

Department of Orchard Plant Genetics and Biotechnology, Institute of Horticulture, Lithuanian Research Centre for Agriculture and Forestry, Babtai, LT-54333 Kaunas, Lithuania; aurelijusstarkus@gmail.com (A.S.); dalia.gelvonauskiene@lammc.lt (D.G.); ingrida.mazeikiene@lammc.lt (I.M.); rytis.rugienius@lammc.lt (R.R.); vidmantas.bendokas@lammc.lt (V.B.); vidmantas.stanys@lammc.lt (V.S.)

* Correspondence: birute.frercks@lammc.lt; Tel.:+370-37-555-253

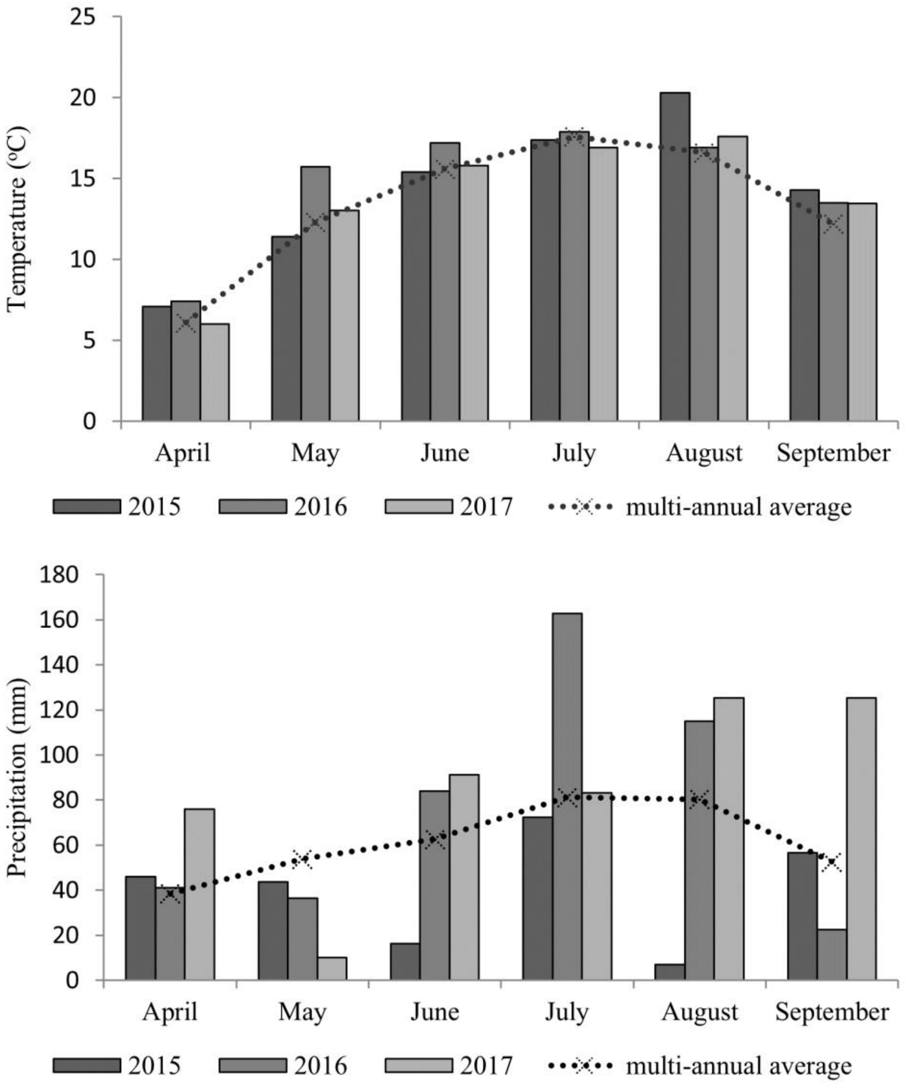

Figure S1. The Meteorological conditions during investigation of apple harvest self-regulation.
